# Osteolysis following PE Wear of a Hastings Head on a Monoblock Hip Stem

**DOI:** 10.1155/2021/9989395

**Published:** 2021-10-07

**Authors:** Sarah Fischer, Valerie Polster, Miriam Ruhr, Robert Hube, Michael M. Morlock

**Affiliations:** ^1^Institute of Biomechanics, Hamburg University of Technology, Denickestraße 15 (K) 21073 Hamburg, Germany; ^2^Orthopaedische Chirurgie Muenchen, OCM-Clinic Munich, Steinerstraße 6, 81369 Muenchen, Germany

## Abstract

We report a case of extended osteolysis, requiring a third revision of the left hip in an 85-year-old man 46 years after index operation. Major polyethylene (PE) wear occurred due to a missmatched combination of a bipolar Hastings head with a PE liner and head damage of the originally maintained stem. This case demonstrates that bipolar heads should not be used with PE cup liners since the respective bearing diameters cannot be guaranteed to match due to missing specifications. Furthermore, putting a Hastings head on an already damaged head of the stem should be omitted and rather the stem should initially be revised.

## 1. Introduction

With a younger age at primary total hip replacement (THR), increased patient activity, and life expectancy, the frequency of revision THR will continue to increase. Fifteen years after primary hip replacement, about 7% of hips had undergone revision surgery [1]. Dependent on the specific case, hip revisions can be more or less complicated, but the procedure should only be as invasive as required. As a consequence, implant components are preserved if possible. In case of aseptic loosening of the acetabular component, which is a frequent reason for hip revision, the femoral stem is often well fixed and therefore should be maintained [1]. Surgeons might choose an implant combination beyond the intended use by the manufacturer for the benefit of a less invasive procedure for the patient. The same applies to situations without information about the implants in situ or the nonavailability of replacement parts, which can also lead to a possibly dangerous off-label combination of components. Isolated cup revisions leaving a monoblock stem with a small head, such as Charnley-type implants, in situ present a challenge. Cup liners with small diameters are not available anymore. Hastings bipolar heads used on a monoblock stem with a 22.225 mm femoral head diameter, combined with a standard acetabular cup liner, have shown promising initial results [2]. Bipolar heads, which offer a higher range of motion, are usually utilized in cases of femoral neck fractures or necrotic femoral heads with intact acetabular cartilage, where the less invasive hemiarthroplasty is often favored over THR.

Although Hastings bipolar heads in an off-label combination of a monoblock stem with a small femoral head and standard acetabular cup liner seem to perform well in some patients [2], we present such a case with a failed Hastings head.

## 2. Case Presentation

**Figure 1:**
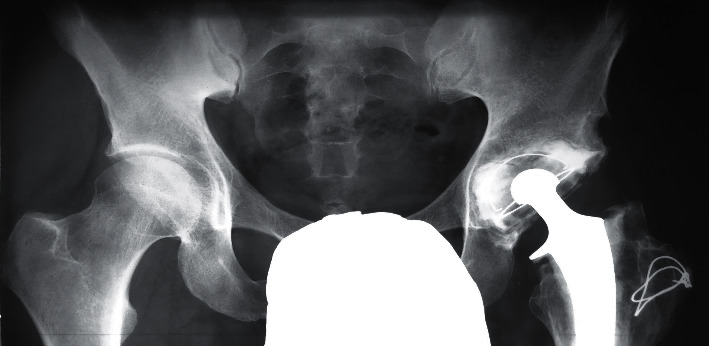
X-ray after primary total hip replacement (1975).

**Figure 2:**
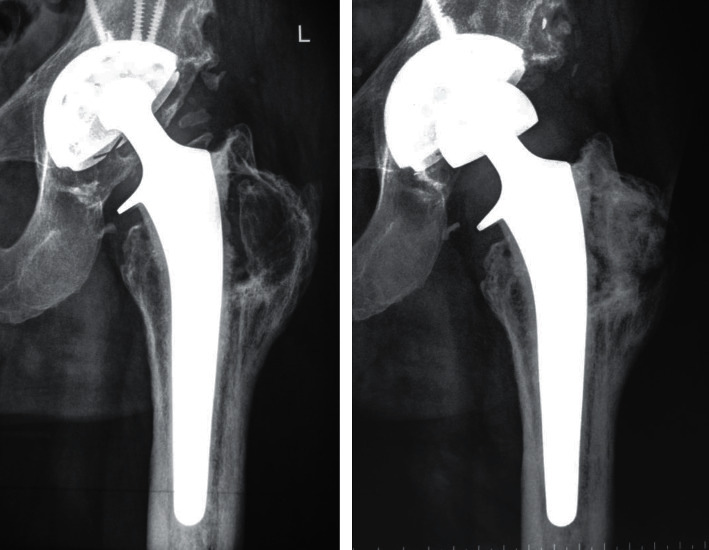
X-ray prior to the second revision showing major osteolysis of the greater trochanter due to PE insert wear (a) (2009); situation after the second revision surgery, in which the greater trochanter was bone grafted, screws were removed, and a 36 mm PE liner was cemented into the uncemented cup (b) (2012).

An 85-year-old male had a conversion osteotomy for dysplasia of his left hip in 1967. Due to increasing arthritis, the hip was replaced in 1975 implanting a collared cemented monoblock stem (Sheehan-Howmedica) with a nominal 22.225 mm head and a cemented PE cup (Figure 1). In 1987, the PE cup was revised due to loosening and converted to an uncemented Harris-Galante cup (Zimmer Biomet, Warsaw, USA) with a PE liner. In 2010, the PE cup liner was revised due to major osteolysis of the greater trochanter (Figure 2). The greater trochanter was bone crafted, some screws were removed, and a 36 mm PE liner was cemented into the existing well-fixed cup and combined with a 36 mm Hastings bipolar head, articulating on the original stem. The histology showed no acute inflammation.

**Figure 3:**
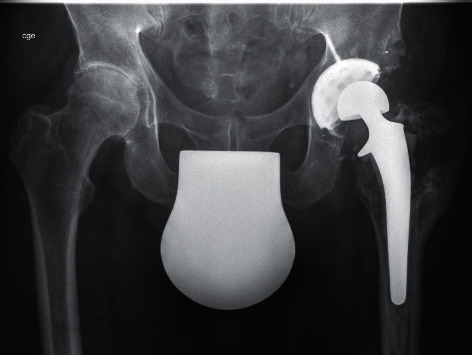
X-ray prior to the third revision surgery (2020).

**Figure 4:**
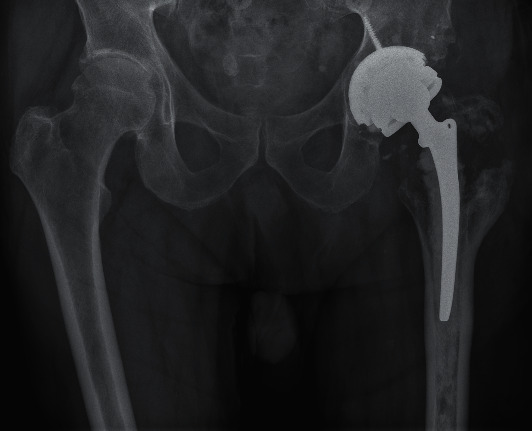
X-ray after the third revision surgery (2020).

**Figure 5:**
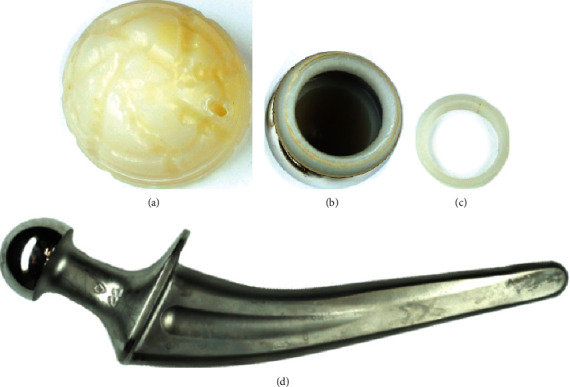
Explanted cup liner (a), bipolar head (b) with a constraining ring (c), and the monoblock stem (d).

The patient presented early 2020 with inguinal pain radiating to the thigh. The diagnosis revealed major osteolysis of the proximal femur with disconnection of the greater trochanter in a fibre-stable situation, in combination with chronical proliferative synovitis, hypertrophic scarring, and trochanteric bursitis. These findings led to a third revision (Figure 3). The monoblock stem was revised to a cemented Weber stem (size F 80) (Figure 4). The cemented PE cup liner was removed using a screw, and a bipolar Avantage cup (Zimmer Biomet, Warsaw, USA; size 50) in combination with a 28 mm ceramic head (M) was cemented into the still well-fixed uncemented Harris-Galante cup from the primary implantation, which was the only component maintained during revision (Figure 5). Additionally, a synovectomy, arthrolysis, necrectomy, and scar excision were performed.

A coordinate measuring machine (CRYSTA-Apex S574, Mitutoyo, Takatsu-ku, Japan; accuracy 3 *μ*m) was used to determine wear at the bearing articulations, and the roughness [3] was examined using a laser microscope (VK-X 150, Keyence, Osaka, Japan; 20x lens). The bipolar head was disassembled into the inner PE and outer metal part after the coordinate measurements.

**Figure 6:**
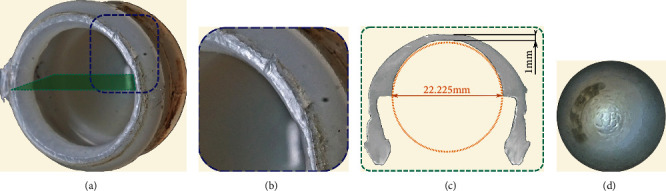
Disassembled PE liner of the bipolar head (a) with signs of stem impingement around the opening (b); a 22.225 mm (nominal diameter) sphere fitted into the cross section in the supposed unworn position (c); imprinting of the bipolar head metal surface on the bottom, indicating cold flow of the PE (d).

**Figure 7:**
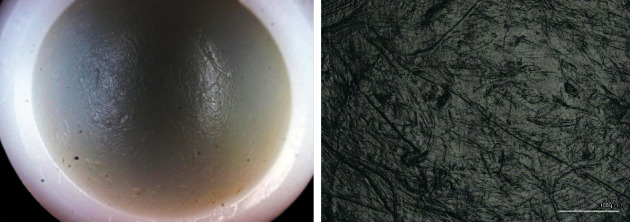
(a) View of the articulating surface of the PE cup liner with multidirectional scratches and (b) pitting magnification of the local surface damage.

**Figure 8:**
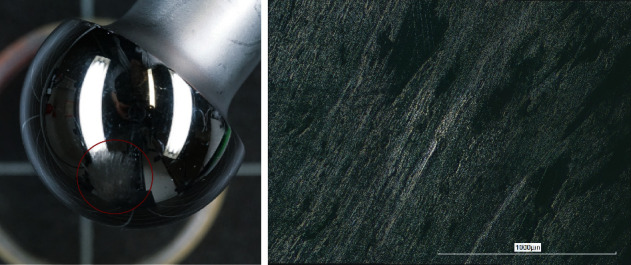
(a) Heavily scratched area of the head with (b) magnification of the local surface damage.

At the pole, the bipolar inner PE liner was worn down to a thickness of only 1 mm (Figure 6(c)). The device identification markings on the inside of the bipolar metal surface were imprinted into the bottom of the PE liner (Figure 6(d)). The rim of the PE head liner showed signs of stem impingement (Figure 6(b)). Dried blood had penetrated between the outer metal and the inner PE liner. The articulating surface of the cup liner showed scratches and pitting throughout without extended polished areas indicative of relative movement and wear (Figure 7). The stem exhibited polished areas close to the tip of the stem indicating some movement in the cement mantle (Figure 5(d)). The articulating head showed a roughened area (Figure 8). The undamaged smooth surface area of the head showed a roughness *R*_a_ of 0.139 *μ*m with a maximum peak to valley height *R*_z_ of 1.170 *μ*m. In the roughened area, these values were more than 10 times increased (*R*_a_: 1.888 *μ*m, *R*_z_: 23.956 *μ*m).

**Figure 9:**
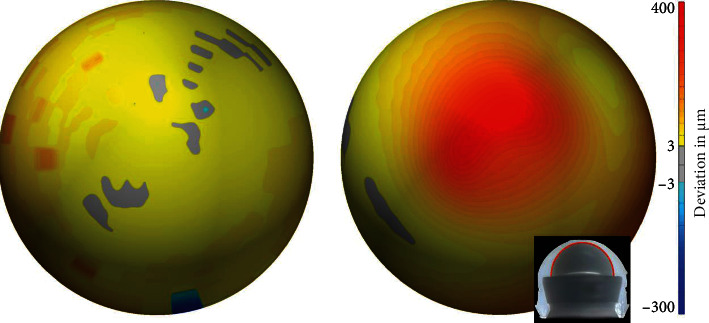
Deviation from a perfect sphere of the PE contact area of the cup liner (a) (*d* = 35.936 mm) and the head liner (b) (*d* = 22.070 mm). Red areas indicate PE wear, and blue areas indicate deposit.

The diameters of the bearing partners were determined by fitting a best-fit sphere to the respective point clouds of the coordinate measurements, excluding worn areas (PolyWorks|Inspector 2019, InnovMetric, Québec, Canada) (Table 1 and Figure 9).

The estimated diameter of the femoral head was 22.194 mm, slightly smaller than the nominal head diameter of 22.225 mm for Charnley-Kerboull's monoblock-type stems. The estimated inner diameter of the PE liner of the bipolar head was 22.070 mm. This results in a negative diametral clearance of -124 *μ*m (“jamming”) on the inner articulation of the bipolar head. The estimated outer diameter of the metal bearing surface of the bipolar head was 36.054 mm. The estimated diameter of the polyethylene cup liner was 35.936 mm. The deformed area from the screw was excluded in the fit. This results in a negative diametral clearance of -124 *μ*m between the outside of the bipolar head and the PE cup liner (“jamming”).

## 3. Discussion

The desired clearance between the head and cup of a ball bearing is usually achieved by manufacturing heads slightly smaller and cups slightly bigger than the nominal diameter. By decreasing the clearance and hence the contact stress, both the friction coefficient and the PE wear rate increase exponentially in a bearing with a metal head and PE liner [4, 5]. In THA bearing components, the initial clearance of PE liners is around 0.1 to 0.3 mm [4]. If there is no clearance between the head and liner, the resulting brake drum effect causes higher friction during motion or even prevents the movement completely.

For the analyzed bipolar head, an outer diameter of 36.054 mm was measured. This is a clear indication that it was not compatible with the 36 mm cup liner. The slightly larger diameter as nominal results from the intended articulation of the bipolar head against native cartilage for which, in contrast to an articulation against a liner, the exact head diameter is not that important since the native cartilage is flexible. The wear pattern of the PE cup liner with mostly pitting and scratching and very little polished areas indicates that there was almost no movement between the bipolar head and the cup liner as a consequence of the measured negative clearance (“jamming”). An originally existing small clearance might have been eliminated due to the swelling of PE in body fluids and a resulting volume growth. During revision surgery, the bipolar head could hardly be moved inside the PE cup liner.

As a consequence, movement of the joint seemed to occur predominantly inside the bipolar head with a small range of motion of only about 30 degrees, which corresponds to less than 50% of a free moving bipolar head. The limited range of motion led to the observed damage at the rim of the PE head liner due to impingement. Hastings bipolar heads are deemed to be compatible with a femoral head diameter of 22.225 mm [2]. In the presented case, the roughened femoral head caused major PE wear and consecutive “carving” of the head into the head liner. The head liner is designed to be slightly thinner centrally by the manufacturer but not to the extent seen in the retrieval [6]. The gap to a circle with the nominal head diameter indicates the amount of PE wear (Figure 6(c)), which most likely induced the osteolysis requiring the revision. The observed negative clearance between the femoral head and the PE head liner, which increased the friction and PE wear rate, was probably caused by swelling of the PE in combination with embedding of the head. Marked PE wear of the cup liner was not observed. The stem movement in the cement mantle, indicated by the polished areas on the stem, might have been enhanced by the higher bearing friction and resulting higher moments.

Dimensional measurements of retrieved PE components have to be treated with great caution due to the material characteristics of PE. Cold flow and fluid uptake influence the measurements to an unknown extent.

## 4. Conclusion

Combining implant components without approval by the manufacturer bear the risk of complications and implant malfunction. Surgeons sometimes have to take this risk to allow a less invasive surgery. In the present case, the combination of a bipolar head with a PE cup liner led to major wear of the PE inside the bipolar head due to the jamming of the bipolar head in the PE cup liner in combination with the roughened area on the monoblock head. Since bipolar heads are not intended to be used against PE cup liners, their actual diameter not necessarily needs to conform to a specific sizing in order to obtain sufficient clearance. This resulted in the observed failure. In the present case, the stem should probably have been revised in the second revision already.

## Figures and Tables

**Table 1:** Diameters and negative diametral clearances of bearing components.

	Labelled [nominal] diameter in mm	Measured diameter in mm	Negative diametral clearance in *μ*m
Femoral head	22 [22.225]	22.194	-124
Head liner	—	22.070	
Outside bipolar head	36	36.054	-108
Cup liner	36	35.936	

## Data Availability

Data is included in the case report.
